# Basal ganglia atrophy–associated causal structural network degeneration in Parkinson's disease

**DOI:** 10.1002/hbm.25715

**Published:** 2021-11-18

**Authors:** Rong Li, Ting Zou, Xuyang Wang, Hongyu Wang, Xiaofei Hu, Fangfang Xie, Li Meng, Huafu Chen

**Affiliations:** ^1^ The Clinical Hospital of Chengdu Brain Science Institute, MOE Key Laboratory for Neuroinformation, High‐Field Magnetic Resonance Brain Imaging Key Laboratory of Sichuan Province, School of Life Science and Technology University of Electronic Science and Technology of China Chengdu China; ^2^ Department of Radiology, Southwest Hospital Third Military Medical University (Army Medical University) Chongqing China; ^3^ Department of Radiology, Xiangya Hospital Central South University Changsha China; ^4^ Sichuan Provincial Center for Mental Health, The Center of Psychosomatic Medicine of Sichuan Provincial People's Hospital University of Electronic Science and Technology of China Chengdu China

**Keywords:** basal ganglia, gray matter, MRI, network degeneration, Parkinson's disease

## Abstract

Parkinson's disease (PD) is a progressive neurodegenerative disease characterized by both motor and non‐motor symptoms. A convergent pathophysiological hallmark of PD is an early selective vulnerability within the basal ganglia circuit. However, the causal interactions between basal ganglia atrophy and progressive structural network alterations in PD remain unaddressed. Here, we adopted voxel‐based morphometry method to measure gray matter (GM) volume for each participant (*n* = 84 PD patients and *n* = 70 matched healthy controls). Patients were first divided into three stages according to the Hoehn and Yahr (H&Y) and the Part III of Unified Parkinson's Disease Rating Scale scores respectively to analyze the stage‐specific GM atrophy patterns. Then, the modulation of early caudate atrophy over other brain structures was evaluated using the whole‐brain voxel‐wise and region‐of‐interest‐wise causal structural covariance network approaches. We found that GM atrophy progressively expands from the basal ganglia to the angular gyrus, temporal areas, and eventually spreads through the subcortical–cortical networks as PD progresses. Notably, we identified a shared caudate‐associated degeneration network including the basal ganglia, thalamus, cerebellum, sensorimotor cortex, and cortical association areas with the PD progressive factors. These findings suggest that the early structural vulnerability of basal ganglia in PD may play a pivotal role in the modulation of motor and non‐motor circuits at the structural level. Our work provides evidence for a novel mechanism of network degeneration that underlies the pathology of PD and may have potential clinical applications in the development of early predictors of PD onset and progress.

## Graphical abstract

Studies have shown that the dopamine depletion within the substantia nigra pars compacta and basal ganglia circuitry is ultimately supposed to be the cause of paucity and slowness of movement in Parkinson's disease (PD). In addition, non‐motor symptoms such as visual disturbances and cognitive impairments have been demonstrated to be associated with early structural damage to the basal ganglia. Here, we examined whether and how the early atrophy of basal ganglia (caudate) drove late‐onset brain structural atrophy in patients with PD. We identified a progressive stage‐specific brain atrophy pattern in PD and demonstrated that the early structural vulnerability of basal ganglia may play a pivotal role in the modulation of motor and non‐motor circuits initiated at the structural level.

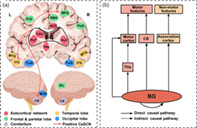

## 1 INTRODUCTION

Parkinson's disease (PD) is a progressive neurodegenerative disease predominantly characterized by motor symptoms, such as tremor, rigidity, akinesia, and postural instability (Jankovic, 2008). Pathophysiological models of PD showed depletion of dopamine within the substantia nigra pars compacta and basal ganglia circuitry in PD (Damier, 1999; Sulzer, 2007). Dopaminergic denervation causes abnormal signal transmission in the nigrostriatal–thalamocortical loop and results in various motor impairments in patients with PD. PD is also associated with various non‐motor symptoms (Seppi et al., 2011; Weerkamp, Nijhof, & Tissingh, 2012) since neurodegeneration worsens during PD. Visual disturbances such as impaired visual acuity, color discrimination, complex visual hallucinations (Archibald, Clarke, Mosimann, & Burn, 2009; Weil et al., 2016), and cognitive impairments including executive dysfunction and problems with working memory (Emre, Ford, Bilgic, & Ey, 2014; Halliday, Leverenz, Schneider, & Adler, 2014) have been reported in patients with PD. The various non‐motor symptoms are likely caused by diffuse pathology and dysfunction beyond the nigrostriatal pathway (Seppi et al., 2011; Weerkamp et al., 2012). Therefore, the relationship of progressive brain damages between basal ganglia circuitry and extra‐nigrostriatal regions needs to be investigated to understand the mechanism of network degeneration in PD.

Advances in structural MRIs have enabled researchers to delineate the progression of structural damage of brain regions in patients with PD. The selective loss of dopaminergic neurons in the nigrostriatal pathway can result in the early structural changes of specific brain regions in PD. Morphometric studies using voxel‐based morphometry (VBM) analysis showed the reduction in the local volume of caudate, putamen, nucleus accumbens, and thalamus in the early stages of PD (Lee et al., 2011; Lee et al., 2014). As the disease progresses, structural damages spread to remote regions and cause neural dysfunction at the network level; such dysfunction may lead to various clinical manifestations and cognitive deficits (Seeley et al., 2006; William, Crawford, & Juan, 2009). Neuroimaging studies showed that structural changes in PD can also extend beyond the brainstem and striatum to other subcortical and cortical regions, such as the temporal and frontal areas (Kassubek, Juengling, Hellwig, Spreer, & Lücking, 2002; Pereira et al., 2012). Recently, it has been shown that moderate‐to‐severe PD patients showed a reduced baseline volume of bilateral caudate nuclei and right hippocampus compared with healthy controls (HC) and mild PD patients (Filippi et al., 2020). Further, several longitudinal structural studies of PD have reported progressive regional atrophy in distributed brain regions, including the basal ganglia, hippocampus, cerebellum, and occipital fusiform gyrus at different stages (Ibarretxe‐Bilbao et al., 2013; Jia et al., 2015; Ramírez–Ruiz et al., 2005). Longitudinal VBM‐based evaluation of the Parkinson variant of multiple system atrophy has shown that short disease duration was associated with progression of atrophy in the striatum, whereas longer disease duration was correlated with increasing atrophy in the cortical areas and cerebellum, suggesting that early degeneration of basal ganglia may drive late‐onset cortical atrophy (Brenneis et al., 2007). Several studies have concluded that basal ganglia is a pivotal node and plays an important role in the pathology of PD. However, most of the previous studies had either small sample sizes, or inadequate methodology (zero‐time lagged), or no healthy‐matched controls. So, the effect of basal ganglia atrophy on other brain structural alterations in PD is still not delineated. Studying the causal relationships of progressive brain structural damage will substantially advance the network degeneration mechanism of PD.

In recent years, structural covariance network (SCN) analysis with neuroimaging data has been used to investigate abnormal structural patterns in patients with neurodegenerative disorders (Alexander‐Bloch, Giedd, & Bullmore, 2013; Brenneis et al., 2007; Evans, 2013; Seeley et al., 2006). SCN measures interregional correlations in cross‐sectional morphometric imaging data. This approach provides a window into the normal developmental relationships and pathological processes between the different parts of the brain. However, one limitation of SCN is that correlation analysis is zero‐time lagged, so possible temporal precedence relationships between different brain regions cannot be reflected. Similar to the correlation analysis used for time‐series data, Granger causality analysis (GCA) is an effective approach for describing the direction of information flow and analyzes whether neural activity in one region precedes that in another region (Goebel, Roebroeck, Kim, & Formisano, 2003; Jiao et al., 2011). Therefore, GCA is widely used in the analysis of resting‐state functional time‐series data to quantify the causal interactions of brain networks (Jiao et al., 2011; Li, Hu, Wang, Liu, & Feng, 2019; Roebroeck, Formisano, & Goebel, 2005). Further, if cross‐sectional morphometric data are associated with temporal information by sequencing morphometric data and disease progression information, then GCA can be a viable approach for characterizing the progressive alterations of the structural brain network. The proposed causal structural covariance network (CaSCN) analysis has been used to study the brain's morphological progression patterns in various neuropsychiatric disorders (Guo et al., 2020; Guo et al., 2021; Jiang et al., 2018; Zhang et al., 2017). Hence, it is an effective analysis for studying the influence of structural alterations in basal ganglia and other regions on PD disease progression.

In this study, we characterized the progressive structural network based on the morphometric data of a cohort of patients with PD (*n* = 84) and age‐ and sex‐matched HC (*n* = 70). We hypothesized that patients with PD would exhibit a progressive brain atrophy pattern. In specific, the basal ganglia atrophy would be the locus of the pathology in the early stages of the disease and would drive the late‐onset structural alterations in spatially distributed subcortical–cortical networks. We examined the stage‐specific progressive pattern of GM volume in patients with PD. Based on two disease progression clinical metrics, we constructed the whole‐brain voxel‐wise and region‐of‐interest (ROI)‐wise CaSCNs to evaluate the causal effects of basal ganglia and other atrophic brain regions.

## 2 MATERIALS AND METHODS

### 2.1 Study setting and participants

The data of all participants with PD and HC were obtained from the Department of Neurology, Xiangya Hospital of Central South University from January 2012 to October 2015. A total of 92 right‐handed patients with PD (mean ± SD = 53.37 ± 10.49 years; 48 men) were diagnosed by at least two or more experienced neurologists who used the UK PD Society Brain Bank criteria (Hughes, Daniel, & Lees, 2001). Patients underwent neurological examinations, including a medical interview. The motor status was evaluated using Part III of the Unified Parkinson's Disease Rating Scale (UPDRS III; Goetz et al., 2008) and Hoehn and Yahr (H&Y) stage (HOEHN, 1967). Some of the patients completed cognitive assessment including the Mini‐mental State Examination (PD, *n* = 72; HC, *n* = 52) and Montreal Cognitive Assessment (MoCA; PD, *n* = 51; HC, *n* = 49). All patients with PD were diagnosed with mild‐to‐severe stages of the disease (Stages 1–5); a high score suggested advanced disease stage. Patients were assessed in an off‐medication state (12 hr after withdrawal of dopaminergic drugs) or a medication state. Exclusion criteria included complicated neuropsychiatric disorders, primary and metastatic tumors, standard contraindications to MRI scanning, any history of drug abuse, incomplete data acquisition (e.g., incomplete MRI scanning and pivotal clinical scores), and poor data quality (e.g., signal artifact). Moreover, none of the patients underwent deep brain stimulation or other brain surgery. Seventy right‐handed HC (mean ± SD = 52.56 ± 10.92 years; 38 men) were recruited from the local community. After all the MRI data of the subjects were matched for age and sex and passed data quality check, 84 patients with PD and 70 HC were enrolled. The demographic characteristics of the participants are provided in Table 1, and the participant‐specific inclusion/exclusion procedure is shown in Figure S1.

**TABLE 1:** Clinical and demographic characteristics of participants

Demographics	PD (*n* = 84)	HC (*n* = 70)	PD vs. HC
	Mean ± SD	Mean ± SD	*p* value
Gender (male/female)	48/36	38/32	.7222^a^
Age (years)	53.37 ± 10.49	52.56 ± 10.92	.3402^b^
Duration (years)	7.49 ± 5.30	—	—
Onset age	47.45 ± 12.06	—	—
Hoehn‐Yahr	2.54 ± 0.95	—	—
UPDRS III	32.99 ± 17.33	—	—
MMSE^c^	26.67 ± 3.57	27.12 ± 4.75	.205^b^
MoCA^c^	22.98 ± 4.41	24.14 ± 5.88	.0297^b^

*Note*: Values are presented as mean ± standard deviation.

Abbreviations: HC, Healthy controls; MMSE, Mini‐mental State Examination; MoCA, Montreal Cognitive Assessment; PD, Parkinson's disease; UPDRS, United Parkinson's Disease Rating Scale.

^a^ *χ* ^2^ test.

^b^ Nonparametric Mann–Whitney tests.

^c^ Partial score missed (MMSE: PD, *n* = 72; HC, *n* = 52; MoCA: PD, *n* = 51; HC, *n* = 49).

### 2.2 Imaging data acquisition

A 3 T GE Signa MR scanner (General Electric Medical Systems) in the Department of Radiology of Xiangya Hospital of Central South University was used to collect T1‐weighted structural imaging data. The acquisition parameters were as follows: repetition time/echo time/inversion time of 7.792 ms/2.984 ms/800 ms; flip angle of 7°; matrix size of 256 × 256; slice thickness of 1 mm; and voxel size of 1 mm × 1 mm × 1 mm. These data were used for analysis after quality assurance.

### 2.3 Data preprocessing

MRI data preprocessing was performed using a morphological processing toolbox (CAT12; http://www.neuro.uni-jena.de/cat/) that was embedded in statistical parametric mapping software (SPM12; https://www.fil.ion.ucl.ac.uk/spm/). Firstly, all images were reoriented to adjust the image origins at the anterior commissure by manual setting after artifact checking and format conversion. Secondly, T1‐weighted images were normalized to Montreal Neurologic Institute (MNI) space and resampled to a volume image resolution of 1.5 mm × 1.5 mm × 1.5 mm with a default template. Then, all images were segmented into three categories—gray matter (GM), white matter, and cerebrospinal fluid. The segmented GM images were nonlinearly modulated to compensate for spatial normalization inference. The nonlinear modulation was essentially corrected for individual differences in brain size. Next, CAT12 was used to display all slices for all images and to calculate the homogeneity of resultant GM maps between subjects. A second manual check was conducted on GM maps with low homogeneity (lower than mean − 2 × SD of the maps from same site) to identify outliers, with the larger value indicating more complete data. Homogeneity was defined as the Pearson correlation coefficient between normalized GM maps of each pair of subjects. Subsequently, the segmented GM images were smoothed by using 4‐mm full‐width‐at‐half‐maximum Gaussian kernel for further analyses.

### 2.4 Voxel‐based morphometric analysis

First, a two‐sample *t* test implemented in SPM12 was performed between the smoothed GM maps of all patients with PD and HC to identify the overall GM regions that had significantly atrophied in patients with PD compared with HC. PD is a progressive disease, so various measures are adopted to evaluate disease severity over its course. The H&Y stages (HOEHN, 1967; Zhao et al., 2010) and UPDRS (Goetz et al., 2008) are two of the most commonly and widely used approaches for estimating PD severity and clinical symptoms. These scales provide systematic and comprehensive descriptions of progressive patterns of the disease. Therefore, we used both measures to evaluate PD disease progression at different stages of the disease. For H&Y stages, the scale is a simple staging assessment that evaluates the severity of overall parkinsonism dysfunction based on bilateral motor involvement and the compromise of gait and balance. Given its historic stature, the scale has been used as a gold standard for a generally accepted clinical translation to mild (Points 1–2), moderate (Point 3), and severe (Points 4–5) levels (Martinez‐Martin, 2010). The original 5‐point scale (Points 1–5) was subsequently modified to a 7‐point scale that included Stages 1.5 and 2.5 in the 1990s (Goetz, et al., 2004). Since the symptoms described by Point 2.5 (mild bilateral disease with recovery on pull test) in this version are much more consistent with Point 3 (mild‐to‐moderate bilateral disease, some postural instability, and physically independent), we defined it as Stage II in the current study. For UPDRS, the scale is reliable and valid in evaluating impairment and disability. Similar to the quartiles' approach, the UPDRS motor scales (Part III) were coded into three groups using the tertiles' approach (Villar et al., 2011; Zhao et al., 2010) in the current study. The first tertile UPDRS III score is 24, and the second one is close to 41. To estimate the pattern of progressive GM atrophy in PD, all patients were categorized into three subgroups based on H&Y stages (Stage I, H&Y: 1–2, *n* = 32; Stage II, H&Y: 2.5–3, *n* = 41; Stage III, H&Y: 4–5, *n* = 11) and UPDRS III scores (Stage I, UPDRS III: 1–23, *n* = 26; Stage II, UPDRS III: 24–40, *n* = 32; Stage III, UPDRS III: 41–74, *n* = 26). To visualize the paired relationship between H&Y and UPDRS scores of each patient, we plotted the group membership across the three stages with paired points (Figure S2). We calculated Pearson's correlation coefficient to quantify the relationship between the two scales. We demonstrate the consistency between these two approaches for estimating PD progression. The detailed demographic information of the subgroups is included in Tables S1 and S2. The smoothed GM volume maps of each subgroup were compared with those of HC by a two‐sample *t* test. Individual sex, age, and total intracranial volume (TIV) were controlled as covariates in the analyses. Gaussian random field (GRF) theory was performed to carry out a cluster‐level correction for comparisons between multiple groups (minimum *z* > 3.09, and cluster significance was set to *p* < .05 and voxel *p* < .001).

To directly compare structural differences for PD patients with or without dementia, we performed additional analysis (Figure S3). Due to the lack of MoCA scores in some patients, this analysis was only performed in patients with MoCA scores (*n* = 51). As suggested by the previous study (Dalrymple‐Alford et al., 2010), we first evaluated the dementia of PD by adopting a MoCA screening cutoff of 21. Then, VBM analysis was used for whole‐brain statistical GM comparison between patients with dementia (*n* = 12) and those without dementia (*n* = 39).

### 2.5 Voxel‐wise causal structural network analysis

The main objective of the study was to test whether an early atrophied brain region exerts a causal influence over other distributed brain networks in patients with PD. CaSCNs were constructed by sequencing the GM maps of all patients from low to high following the ranks of H&Y and UPDRS III scores. The sequenced data were analogous to time‐series information, which is used for characterizing the progressive structural alterations of PD based on cross‐sectional data. As a region showing early GM atrophy (Figure 2 and Tables S4 and S5), the most atrophied left caudate was selected as the seed by comparing the patients with PD and HC (MNI coordinates: −17, −3, 20, radius = 5 mm) to construct CaSCNs for PD. The average GM values within the left caudate were extracted from sequenced morphometric data and used as the pseudo‐time‐series. Signed‐path coefficient GCA disposed with an fMRI toolbox (REST; http://www.restfmri.net; Zang, Yan, Dong, Huang, & Zang, 2012) was performed on a voxel‐wise basis for all the voxels in the mask of the brain areas with GM. Based on temporal precedence, GCA was first proposed to test whether the past value of a time course correctly predicts the current value of another. A directional GC value from region X to region Y was inferred if the past value of time course X helped predict the current values of the time‐series of region Y, that is, X had GC influence on Y (Granger, 1969; Jiao et al., 2011; Zang et al., 2012). CaSCN (applying GCA on the pseudo‐time‐series morphometric data through sequencing) can accordingly estimate the causal effect of the atrophy of a brain region on other regions (Guo et al., 2020; Guo et al., 2021; Wu et al., 2012; Zhang et al., 2017). GCA can describe the causal effects caused by basal ganglia atrophy by sequencing morphometric data (H&Y and UPDRS III scores). The seed region showed GM atrophy in patients with PD. The positive signed‐path coefficient from X to Y indicates that the GM volume decreases in the region of Y lags behind the seed atrophy of X. Given that the selective vulnerability of basal ganglia is an important pathophysiological hallmark of PD, only GC values of X to Y (from the caudate to the whole brain) were utilized. Individual sex, age, and TIV were regressed as covariates in CaSCN analyses. The GC map was z‐score‐transformed to present statistical significance, a GRF correction was applied (minimum z > 3.09, and cluster significance was set to *p* < .05 and voxel *p* < .001). Finally, we performed conjunction analysis to identify spatial similarity between H&Y and UPDRS III‐sequenced CaSCNs.

### 2.6 ROI‐wise causal structural network analysis

ROI‐wise causal network analysis was performed on the sequenced morphometric data to investigate the relationships of temporal precedence among brain regions. We hypothesized that PD shows a common caudate‐associated degeneration network based on the progressive disease factors of H&Y stages and UPDRS III scores. Thus, ROIs were extracted from the abovementioned overlapped CaSCN maps to determine the existence of a causal link or causal loop between basal ganglia and specific subcortical and cortical networks. Signed‐path coefficient GCA was performed to conduct an ROI‐to‐ROI causal network measurement of interregional GC relationships among ROIs. Sign‐GC values were *z*‐distribution‐transformed, and the results were considered statistically significant if *p* < .05. Only the GC values of X to Y were assessed.

## 3 RESULTS

### 3.1 Overall GM atrophy pattern in PD

**FIGURE 1:**
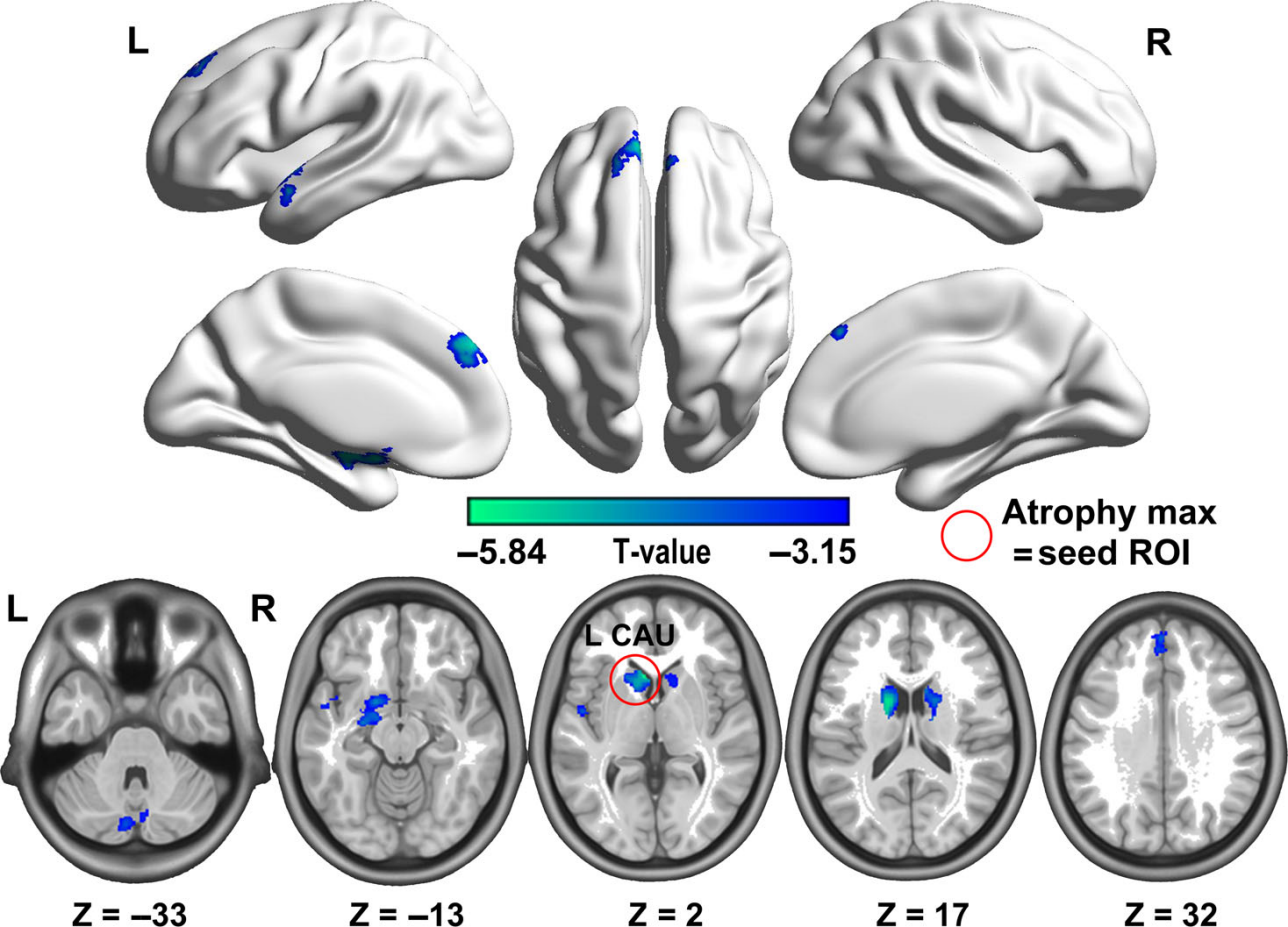
Overall gray matter atrophy pattern in patients with PD. Compared with the healthy controls, patients with PD displayed significantly reduced gray matter volume in the bilateral caudate, superior temporal lobe, left superior frontal gyrus, hippocampus, and cerebellum. The most atrophied area is in the left caudate. GRF was used for cluster‐level correction for whole‐brain multiple comparisons (minimum z > 3.09, cluster significance was set to *p* < .05, and voxel *p* < .001). L, Left; R, right; ROI, region of interest

We conducted a whole‐brain statistical GM comparison between patients with PD and HC. Compared with HC, patients with PD displayed significantly decreased GM volume in the following: the basal ganglia network, including the caudate and putamen; limbic hippocampal gyrus; cerebellum; and cortical regions, such as the medial superior frontal gyrus, superior frontal gyrus, and superior temporal lobe (minimum *z* > 3.09, cluster significance was set to *p* < .05 and voxel *p* < .001, GRF corrected; shown in Figure 1). The details of widespread subcortical–cortical atrophy in patients with PD are summarized in Table S3. No significantly increased GM volume was found in PD.

### 3.2 Progressive stage‐specific GM atrophy patterns in PD

**FIGURE 2:**
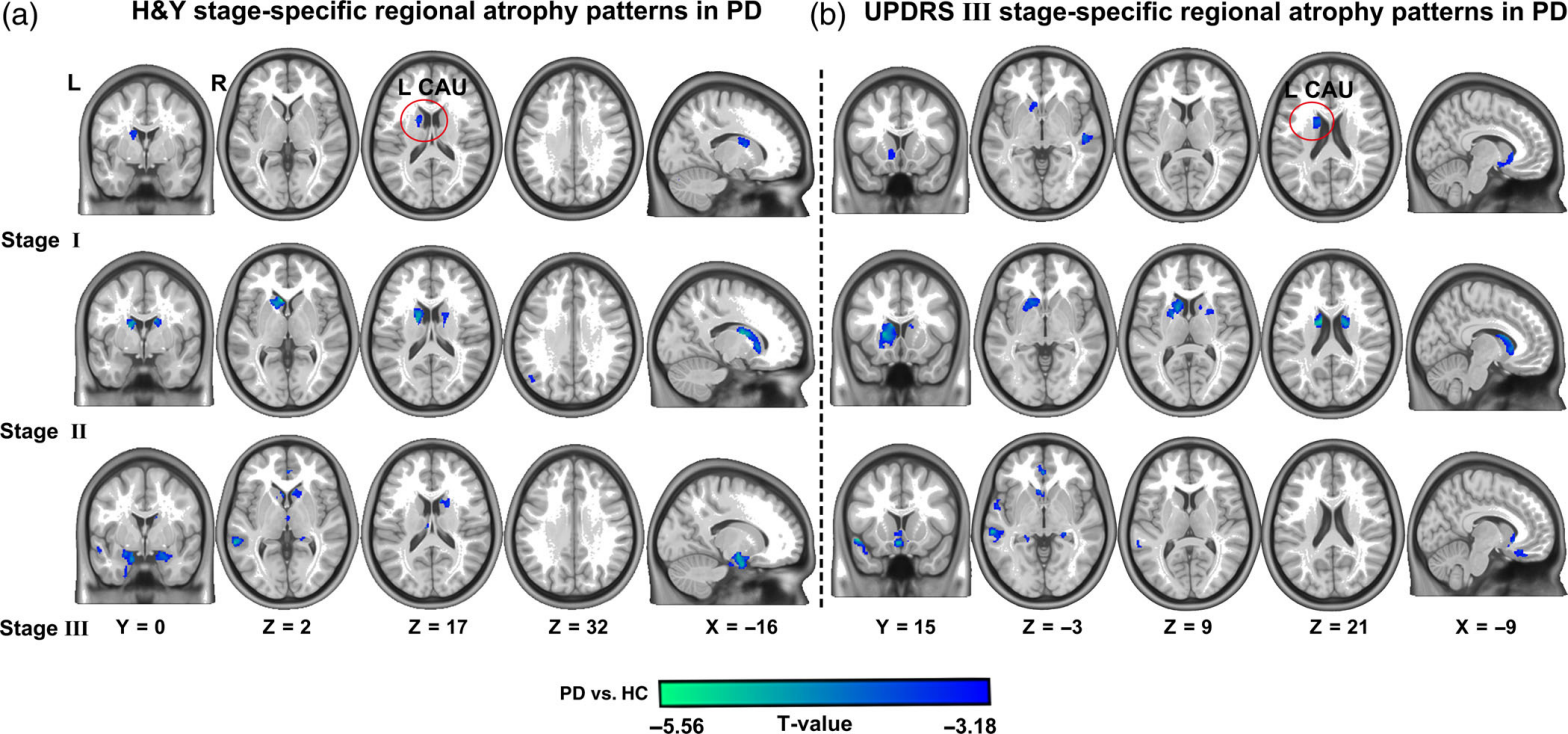
Progressive patterns of stage‐specific gray matter atrophy in patients with PD. All patients with PD were categorized into three subgroups per the H&Y (a) and UPDRS III (b). Similar stage‐specific brain atrophy patterns were observed in these two different progressive factors. Patients with PD were associated with early atrophy in the left caudate (Stage I). The pattern of atrophy progressively expanded to the bilateral basal ganglia (putamen) and angular gyrus (Stage II) and enveloped cerebellum and more distributed subcortical and cortical regions (Stage III). GRF was used to carry out cluster‐level correction for whole‐brain multiple comparisons (minimum z > 3.09, cluster significance *p* < .05, and voxel significance *p* < .001). HC, Healthy controls; H&Y, Hoehn and Yahr; L, left; PD, Parkinson's disease; R, right; UPDRS, United Parkinson's Disease Rating Scale

We addressed how brain atrophy progressed during PD. To characterize the progressive patterns of GM atrophy, the patients were grouped into three stages based on H&Y and UPDRS III scores. H&Y score‐specific subgroup comparisons revealed that patients with PD exhibited early atrophy in the left caudate (Stage I). With progressing PD, the pattern of atrophy progressively expanded to the left putamen, angular gyrus, and right caudate (Stage II). Additional distribution regions, such as the bilateral hippocampus, thalamus, left middle and superior temporal gyrus, and medial orbitofrontal cortex, were affected in later stages of the disease (Stage III, shown in Figure 2a; minimum *z* > 3.09, *p* < .001, GRF corrected). We observed similar brain atrophy patterns for the progressive factor of UPDRS III scores (Figure 2b, minimum *z* > 3.09, *p* < .001, GRF corrected). Compared with HC, patients with PD displayed significantly decreased GM volume in the left caudate and right superior temporal gyrus (Stage I). Atrophy progressed to the right caudate and putamen (Stage II) and then to the bilateral medial orbital frontal cortex, parahippocampal gyrus, temporal lobe, and cerebellum (Stage III). Detailed stage‐specific brain atrophy is summarized in Tables S4 and S5.

The VBM analysis between PD patients with and without dementia revealed that the two groups did not differ significantly in the basal ganglia and associated degeneration motor network areas. However, PD patients with dementia exhibited significant atrophy in the anterior cingulate cortex and medial superior frontal gyrus (see Figure S3).

### 3.3 Voxel‐wise CaSCNs show a causal effect of early caudate atrophy on distributed GM changes

**FIGURE 3:**
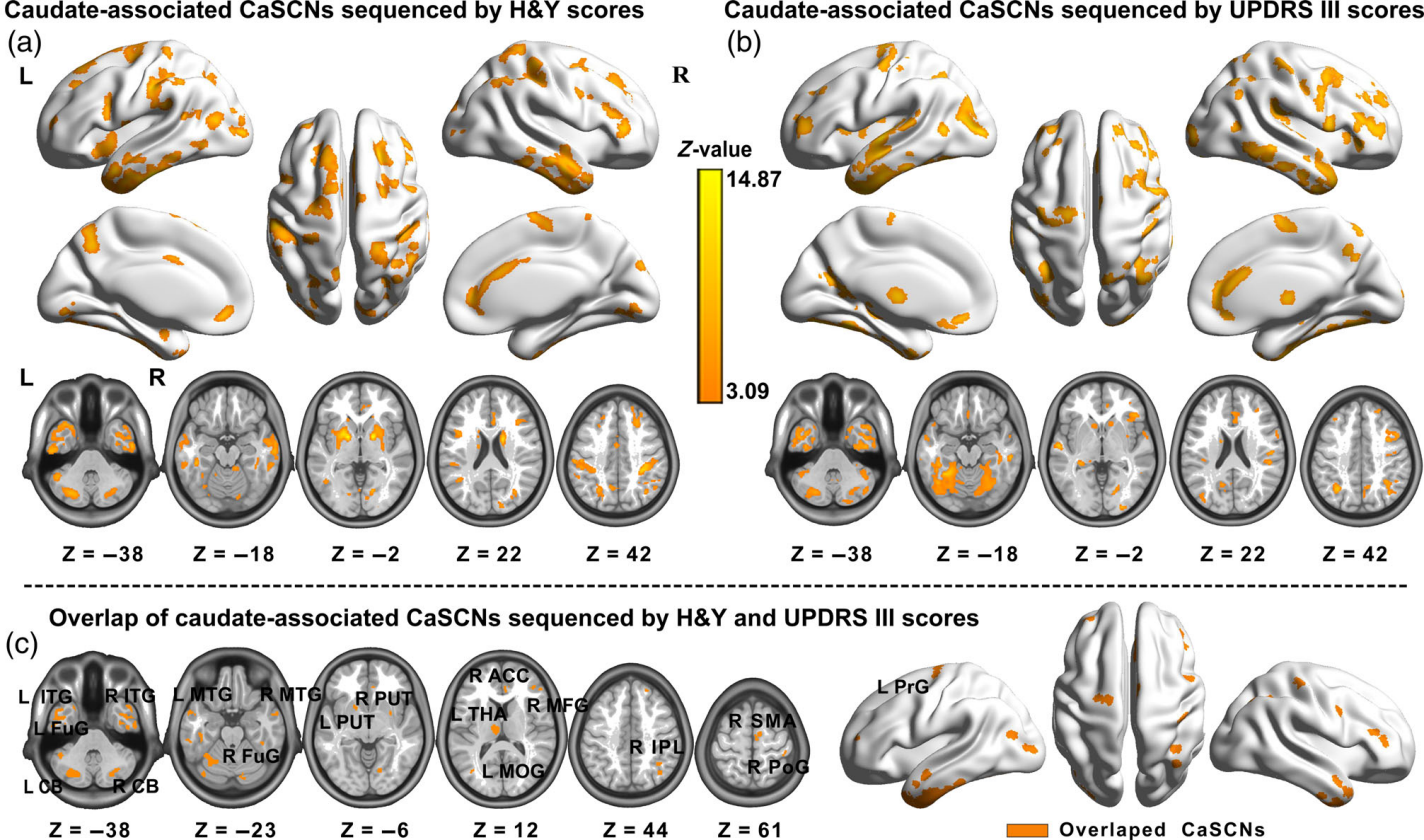
Caudate‐associated voxel‐wise causal networks of structural covariance (CaSCNs) in patients with PD. CaSCNs were constructed by using Granger causal analysis to sequence gray matter maps according to progressive disease factor of H&Y (a) and UPDRS III (b) scores. We identified the shared caudate‐associated positive CaSCNs, including the striatal regions (putamen), fusiform gyrus, inferior and middle temporal gyruses, angular gyrus, inferior parietal lobule, primary motor cortex, anterior cingulate cortex, and cerebellum (c). All brain clusters were GRF corrected by using a height threshold of cluster significance *p* < .05 and voxel significance *p* < .001 (minimum z > 3.09). ACC, Anterior cingulate cortex; CB, cerebellum; FuG, fusiform; H&Y, Hoehn and Yahr; IPL, inferior parietal lobule; ITG, inferior temporal gyrus; L, left; MFG middle frontal gyrus; MOG, middle occipital gyrus; MTG, middle temporal gyrus; PD, Parkinson's disease; PoG, postcentral gyrus; PrG, precentral gyrus; PUT, putamen; R, right; SMA, supplementary motor area; THa, thalamus; UPDRS, United Parkinson's Disease Rating Scale

To verify whether early atrophy of basal ganglia (caudate) drove late‐onset brain atrophy in patients with PD, we implemented CaSCNs through seeding at the left caudate. Figure 3a shows the progressive factor of H&Y stages (minimum z > 3.09, *p* < .001, GRF corrected, Table S6). The bilateral basal ganglia (caudate, putamen, and pallidum), thalamus, cerebellum, middle occipital gyrus, fusiform gyrus, lateral temporal cortex, primary and secondary motor cortex, and prefrontal cortex showed significant positive GC effects. The atrophy of these regions with reduced GM volume lagged behind the left caudate, thereby implying that structural atrophy in these areas could be driven by the seed. Similarly, voxel‐wise CaSCNs were constructed per UPDRS III scores (minimum z > 3.09, *p* < .001, GRF corrected, Figure 3b and Table S7). Positive GC values were found in the bilateral caudate, putamen, thalamus, cerebellum, middle occipital gyrus, fusiform gyrus, middle and inferior temporal gyrus, primary and secondary motor cortex, inferior parietal lobule, prefrontal cortex, and anterior cingulate cortex. We further performed a conjunction analysis to identify the shared caudate‐associated CaSCNs from Figure 3a and b. This approach revealed the common positive CaSCNs of the subcortical and cortical regions, including the bilateral basal ganglia (putamen), cerebellum, thalamus, occipitotemporal cortex (fusiform gyrus, middle occipital gyrus, and inferior and middle temporal gyruses), inferior parietal lobule, medial and lateral prefrontal cortex (anterior cingulate cortex, middle frontal gyrus), and motor cortex (precentral gyrus, postcentral gyrus, and supplementary motor cortex), as shown in Figure 3c.

### 3.4 ROI‐wise CaSCNs reveal interregional causal structural networks

**FIGURE 4:**
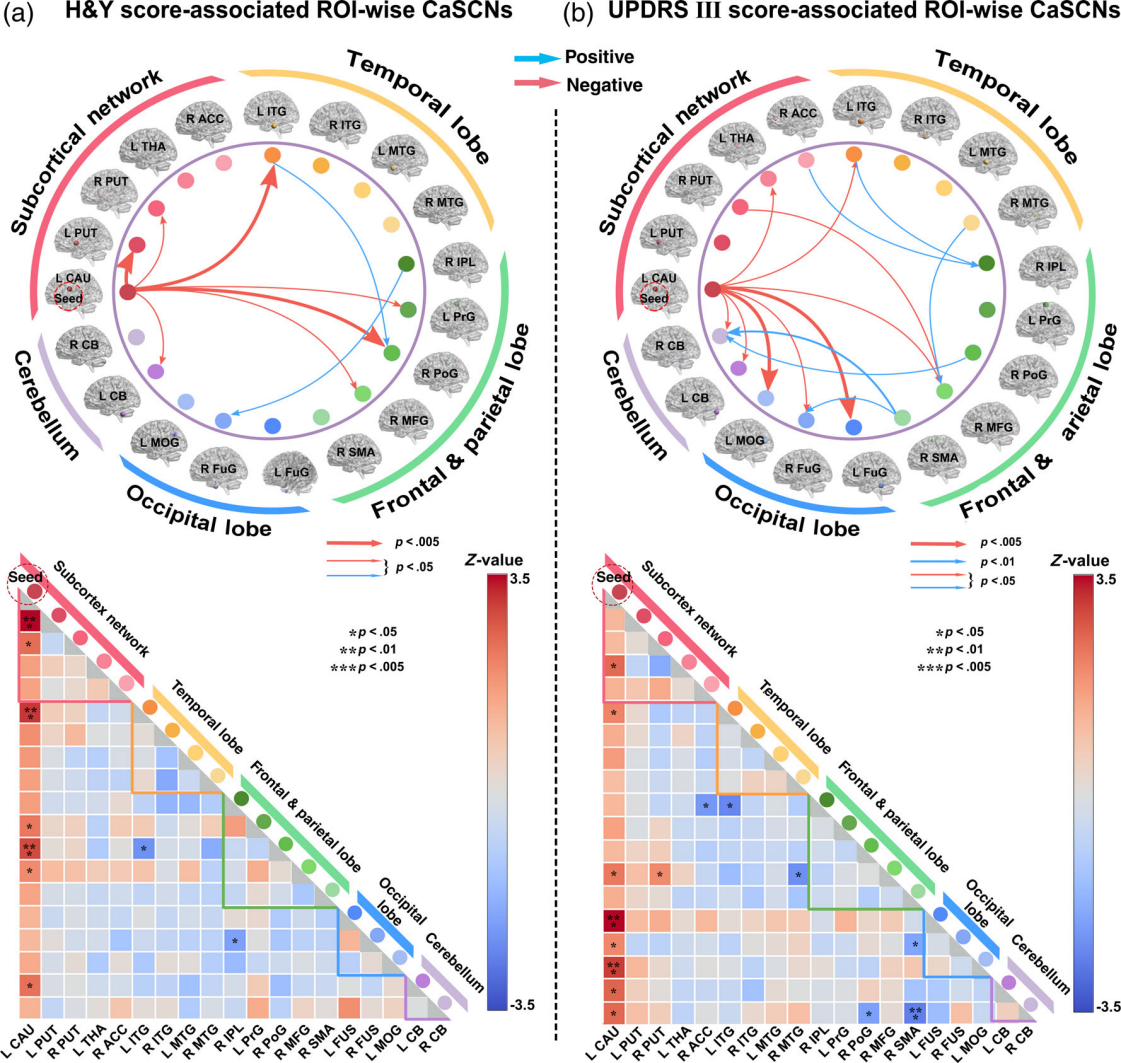
ROI‐wise causal networks of structural covariance (CaSCNs) in patients with PD. Granger causality analysis was performed to construct ROI‐wise CaSCNs based on H&Y (a) and UPDRS III (b) scores to characterize the interregional structural causal network among ROIs. Arrows with warm and cool colors in the wheel represent positive and negative GC values, respectively. The left caudate, as the hub of the directional network, has extensive positive causal effects on the distributed networks, such as the subcortical network, temporal, frontoparietal, and occipital networks, as well as the cerebellum (*p* < .05). ACC, Anterior cingulate cortex; CAU, caudate; CB, cerebellum; FuG, fusiform; H&Y, Hoehn and Yahr; IPL, inferior parietal lobule; ITG, inferior temporal gyrus; L, left; MFG middle frontal gyrus; MOG, middle occipital gyrus; MTG, middle temporal gyrus; PoG, postcentral gyrus; PrG, precentral gyrus; PUT, putamen; R, right; ROI, region of interest; SMA, supplementary motor area; THa, thalamus; UPDRS, United Parkinson's Disease Rating Scale

ROI‐wise CaSCNs revealed the directional relationship of temporal precedence among ROIs extracted from the overlapped voxel‐wise CaSCN maps and demonstrated the interregional structural causal network in PD. The ROI‐wise CaSCNs, sequenced by H&Y stages, revealed that the left caudate had extensive positive causal influences within the basal ganglia network (bilateral putamen), motor and prefrontal areas (precentral, postcentral, and middle frontal gyruses), inferior temporal gyrus, and cerebellum (Figure 4a, *p* < .05, uncorrected). The ROI‐wise CaSCNs, sequenced by UPDRS III scores, showed positive GC values principally from the left caudate to the thalamus, inferior temporal gyrus, the middle frontal gyrus, visual cortex (middle occipital gyrus and fusiform gyrus), and cerebellum (Figure 4b, *p* < .05, uncorrected). The common ROI‐wise CaSCNs were observed from the left caudate to the left inferior temporal gyrus, right middle frontal gyrus, and cerebellum.

### 3.5 The integrative basal ganglia atrophy–associated causal pathways

**FIGURE 5:**
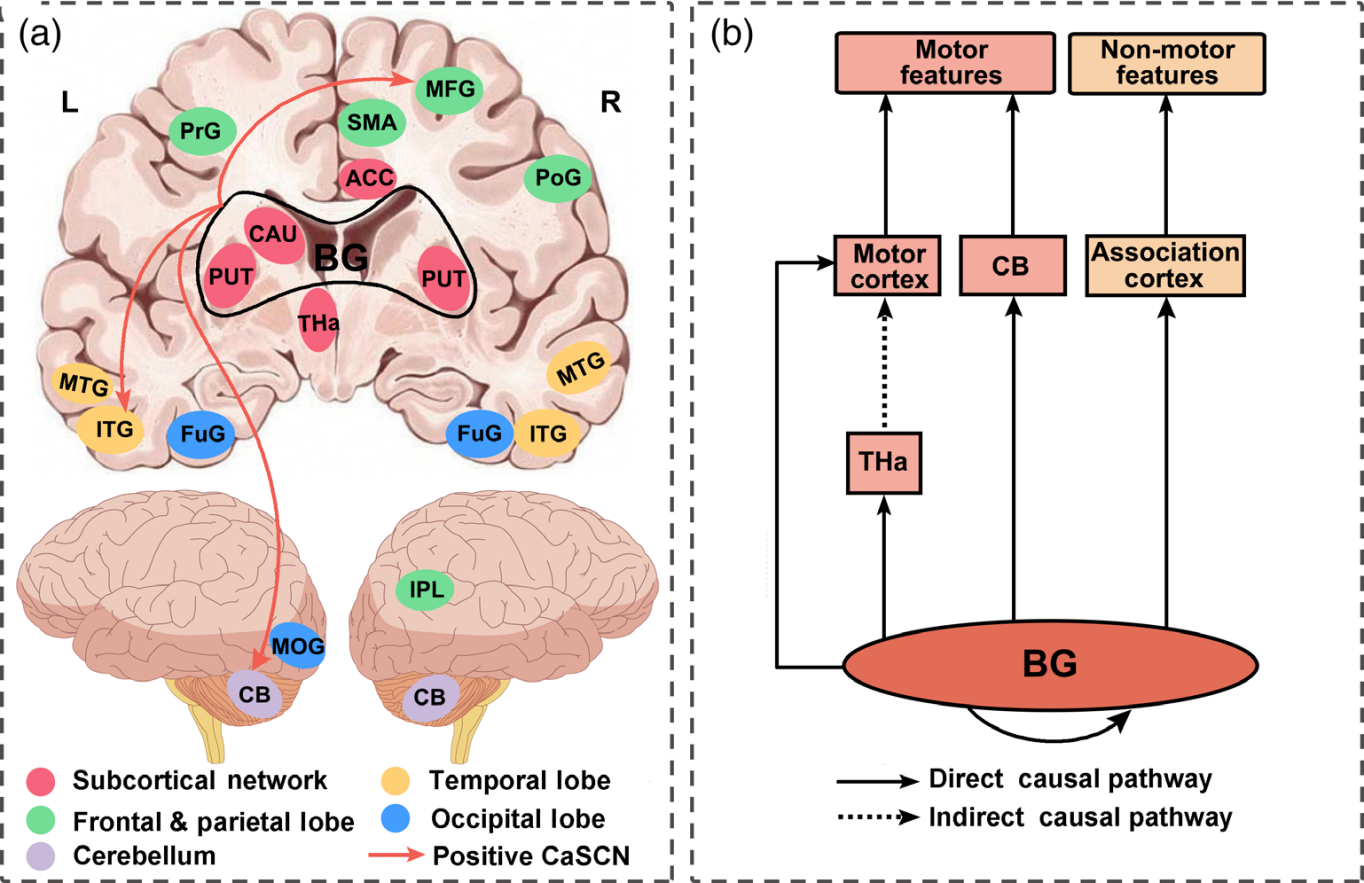
Schematic causal pathways of the spread of GM volume with different brain structures associated with basal ganglia atrophy based on our study (direct) and the previous research (indirect). (a) Regions showed the location of the common caudate‐associated CaSCNs (Figure 3c) constructed with the progressive disease factors of H&Y and UPDRS III scores. The arrows with warm color represent the results of overlapped ROI‐wise CaSCNs (Figure 4). (b) Summary diagram of basal ganglia (BG)‐associated causal structural network related to the motor and non‐motor features of PD. ACC, Anterior cingulate cortex; CaSCN, causal structural covariance network; CB, cerebellum; CAU, caudate; FuG, fusiform; GM, gray matter; H&Y, Hoehn and Yahr; IPL, inferior parietal lobule; ITG, inferior temporal gyrus; L, left; MFG middle frontal gyrus; MOG, middle occipital gyrus; MTG, middle temporal gyrus; PoG, postcentral gyrus; PrG, precentral gyrus; PUT, putamen; R, right; ROI, region of interest; SMA, supplementary motor area; THa, thalamus; UPDRS, United Parkinson's Disease Rating Scale

A schematic causal pathway of the spread of GM volume atrophy in different brain regions associated with basal ganglia was summarized (Figure 5) by combining our results (direct) and previous studies (indirect; Baev et al., 2002; Braak et al., 2003; Schapira, Chaudhuri, & Jenner, 2017; Surmeier, Obeso, & Halliday, 2017). The distributed structural network progression of PD included the basal ganglia, thalamus, motor cortex, cerebellum, and association cortex. This progression pattern underlies the motor and non‐motor features of PD with putative causal circuits, as depicted in Figure 5b. Overall, the basal ganglia in these interactions may play a pivotal role in the modulation of motor (thalamus, cerebellum, and motor cortex) and non‐motor (associate cortex) circuits (initiated at structural levels) by reinforcing the current causal patterns via the direct pathway and inducing clinical characteristics via the indirect pathway.

## 4 DISCUSSION

We identified a progressive stage‐specific brain atrophy pattern and demonstrated a basal ganglia‐associated causal degeneration network in patients with PD. Early selective structural vulnerability occurred in the left caudate. Then, as the disease progressed, damage spread to the angular gyrus and temporal areas and throughout spatially distributed subcortical–cortical networks. By applying whole‐brain voxel‐wise and ROI‐wise CaSCNs on the sequenced cross‐sectional morphometric maps based on different disease progression measures, we revealed a common caudate‐associated degeneration network that included the subcortical basal ganglia, thalamus, and cerebellum, as well as motor cortex, occipitotemporal cortex, and higher‐order association cortex. These findings suggest that the early vulnerability within the basal ganglia is central to the disruption of various structural network alterations in PD.

Consistent with findings in previous studies that aimed to characterize the structural changes of PD (Feldmann, Illes, Kosztolanyi, Illes, & Nagy, 2008; Ghaemi, Hilker, Rudolf, Sobesky, & Heiss, 2002; Lee et al., 2014; Xu et al., 2020; Xu, Yang, Hu, & Shang, 2016), we found that patients with PD showed widespread GM reductions in basal ganglia and various subcortical–cortical structures. As a progressive disease, one of the important issues to address is whether the pathology of PD simultaneously targets all the basal ganglia and extrastriatal regions or the different structures differ in vulnerability. Recent longitudinal studies have reported progressive regional atrophy in several brain regions, including the basal ganglia, hippocampus, thalamus, occipital fusiform gyrus, and frontal cortex by comparing PD at different time points in one patient (Gee et al., 2017; Ibarretxe‐Bilbao et al., 2010). However, evaluating whether the progressive GM patterns are specific to patients with PD was difficult because no healthy matched controls were included. In the current study, we used two of the most commonly used clinical metrics (H&Y scales and UPDRS scores) to characterize the structural progression of patients with PD by comparing GM maps at different stages with age‐ and gender‐matched HCs. Interestingly, we observed a common stage‐specific progressive GM atrophy pattern, in which the morphological changes of the left caudate existed in the early disease stages, whereas spatially distributed subcortical–cortical network alterations were highly prominent in the later stages. Our findings broadly support the Braak staging model for the progression of Lewy‐type alpha synucleinopathy pathology (Braak et al., 2003), which begins in the subcortical nucleus and gradually progresses to high‐order sensory association areas and prefrontal cortex during the progression of PD. The relatively early involvement of basal ganglia atrophy can be because of the neural loss in the nigrostriatal pathway in early disease stages. The spread of atrophy from the basal ganglia to the thalamus, cerebellum, and cortical regions in later stages is likely related to the pathological spread through anatomically coupled networks.

In addition to providing supporting evidence for the anatomical distribution of the damage, we offer a novel perspective on the network degeneration of PD by investigating the potential causal relationships of structural network alterations. We proposed a strategy of CaSCN by assigning “time‐series” on cross‐sectional morphometric data following PD disease progression information. The application of CaSCN can estimate the causal effect of morphometric alteration in one preceding region and predict the disease occurrence in regions in other networks as PD progresses. In contrast to some other neuroimaging technologies for mapping disease progressions, such as structural analysis based on the stage‐specific comparison (Shima, Matsunari, Samuraki, Chen, & Yamada, 2011) and longitudinal investigation (Brenneis et al., 2007), CaSCN is a highly comprehensive and theoretically reliable method for describing the causal relationships of interregional structural damages. Using this approach, we found that the left caudate atrophy showed significant positive causal influence on the bilateral basal ganglia, thalamus, cerebellum, occipitotemporal cortex, primary motor cortex, and the parietal and prefrontal association cortex. Notably, our study showed a core shared caudate‐associated CaSCNs in subcortical and cortical networks from both progressive factors of H&Y stages and UPDRS III scores, thereby suggesting a strong causal influence of caudate atrophy on progressive brain structural alterations. The presence of the CaSCNs was largely consistent with progressive GM atrophy, as revealed by our stage‐specific subgroup comparisons (Figure 2). These results may indicate that during the pathological process of PD‐related damage on brain structures, dopaminergic denervation begins with basal ganglia regions (caudate and putamen), spreads via specific network nodes (e.g., thalamus), and selectively causes damage on distributed networks in different orders (Braak, Del Tredici, et al., 2003; Surmeier et al., 2017). ROI‐based CaSCN further also revealed that the left caudate, as the hub of the directional network, had extensive positive causal effects on other regions, particularly the inferior temporal gyrus, middle frontal gyrus, and cerebellum.

Positive GC values were found in the bilateral putamen, cerebellum, thalamus, and sensorimotor cortex both in the CaSCNs with H&Y stages and UPDRS III scores. These structures are anatomically and functionally projected to basal ganglia circuitry and are involved in modulating or producing motor output (Houk & Wise, 1995; Turner & Desmurget, 2010). These are also selectively vulnerable regions underlying the motor symptoms of patients with PD (Mikell & McKhann, 2010; Wu & Hallett, 2013). In particular, the basal ganglia sustains motor and sensory functions through the modulation and integration of information from the substantia nigra, thalamus, and cortex. The loss of dopaminergic input to the basal ganglia can alter motor control and sensory perception of patients with PD. Many studies have reported GM reductions of basal ganglia, motor cortex, and thalamus in patients with PD (Feldmann et al., 2008; Gee et al., 2017; Ghaemi et al., 2002; Ibarretxe‐Bilbao et al., 2010; Lee et al., 2014; Tessa et al., 2014; Xu et al., 2016). The severity of the motor impairments of PD has been related to cerebellar atrophy and decreased cerebellar–striatal–sensorimotor interactions (Gilat et al., 2017; O'Callaghan et al., 2016). The positive causal effects on the motor circuit suggest that the morphometric alterations follow the caudate atrophy and may be associated with worsening motor symptoms with disease progression.

In addition to the positive causal effects identified in the motor circuit, several cortical regions, including the fusiform gyrus, occipitotemporal cortex, and parietal and prefrontal association areas, also exhibited positive GC values. The fusiform gyrus and inferior parietal lobule are critical for high‐level visual and visuospatial processing. In addition, the decreased GM volume in the right superior and medial frontal gyrus, putamen, and caudate nucleus were also significantly associated with visual attention performance in PD (Pezzoli, Cagnin, Antonini, & Venneri, 2019). The prefrontal and anterior cingulate cortexes are involved in cognitive and emotional processes. The structural damage of these regions in patients with PD have been reported in previous studies (Brenneis et al., 2007; Herman, Rosenberg‐Katz, Jacob, Giladi, & Hausdorff, 2014; Lee et al., 2014; Pereira et al., 2012) and in our later stage‐specific results (Figure 2). We also identified significantly larger GM atrophy in the anterior cingulate cortex and medial prefrontal cortex in PD patients with dementia compared with patients without dementia. This observation suggests that the non‐motor symptoms of PD, such as cognitive impairments, may be associated with the structural atrophy of cognitive networks. We speculated that the spatially distributed cortical CaSCNs may be related to the clinical non‐motor features of PD, such as visual disturbances, cognitive decline, or psychiatric impairment (Schapira et al., 2017). The degeneration of the basal ganglia may be an important role in the modulation of motor as well as non‐motor circuits at structural levels.

Several limitations of the current study should be noted. First, given the inherent low proportion of PD patients in later H&Y stages (Coelho et al., 2010; Coelho & Ferreira, 2012; Enders et al., 2017) and the difficulty of head motion control in late‐stage patients during MRI data acquisition, the sample size of patients with late‐stage disease is relatively small in our study. Secondly, although age and TIV were regressed as covariates in VBM and CaSCN analysis, ruling out such inherent differences among individuals is difficult. Our study was also based on patients with non‐first‐episode PD. Thus, our findings might have been confounded by long‐term treatment with dopaminergic medications. Also, as a chronic neurodegenerative disorder, various measures are adopted to evaluate disease severity over PD's course. Here, stage‐specific comparisons were performed by categorizing patients into subgroups based on only H&Y and UPDRS III scores, so it is necessary to take into account the non‐motor symptoms, such as cognitive impairments, to define the severity of the disease in future studies. Finally, applying temporal information to cross‐sectional morphometric data may not directly reflect the real temporal sequence of illness. Longitudinal studies on large cohorts of patients with PD are necessary to further confirm our findings and to accurately follow brain modifications during disease progression.

We demonstrated the core common caudate‐associated CaSCNs of subcortical–cortical networks based on different progressive information in patients with PD. Our findings suggest that the early selective vulnerability of basal ganglia may play a central role in modulating late‐onset motor and non‐motor circuit damage. This study provides evidence for a novel network degeneration mechanism of PD and may have potential clinical applications and help in the development of early predictors of PD onset and progress.

## CONFLICT OF INTEREST

The authors declare no competing financial interests.

## ETHICS STATEMENT

Our study was reviewed and approved by the institutional ethics committee of Central South University.

## PATIENT CONSENT STATEMENT

All subjects provided written informed consent before the experiment.

## Supporting information


**Appendix** S1: Supporting Information

## Data Availability

The data that support the findings of this study are available from the corresponding author upon reasonable request.
