# Supplementary material for: Basal ganglia atrophy–associated causal structural network degeneration in Parkinson's disease
Source: Hum Brain Mapp. 2021 Nov 18;43(3):1145–56. doi: 10.1002/hbm.25715 (PMC8764481; doi:10.1002/hbm.25715)
Supplement: Supplementary file 1 — Appendix S1: Supporting Information [file HBM-43-1145-s001.doc]

**Online Supplementary Materials For:**

**Basal ganglia atrophy associated causal structural network degeneration in Parkinson’s disease**

Running title：Caudate-associated CaSCNs in Parkinson’s disease

Rong Li^1#^, Ting Zou^1#^, Xuyang Wang^1^, Hongyu Wang^1^, Xiaofei Hu^3^, Fangfang Xie^4^, Li Meng^4*^, and Huafu Chen^1,2*^

^1^ The Clinical Hospital of Chengdu Brain Science Institute, MOE Key Laboratory for Neuroinformation, High-Field Magnetic Resonance Brain Imaging Key Laboratory of Sichuan Province, School of Life Science and Technology, University of Electronic Science and Technology of China, Chengdu, 610054, P.R. China.

^2^ Sichuan Provincial Center for Mental Health, The Center of Psychosomatic Medicine of Sichuan Provincial People's Hospital, University of Electronic Science and Technology of china, Chengdu 611731, China.

^3^ Department of Radiology, Southwest Hospital, Third Military Medical University (Army Medical University), Chongqing, 400038, China.

^4^ Department of Radiology, Xiangya Hospital, Central South University, Changsha, 410008, P.R. China.

^#^ Rong Li and Ting Zou contributed equally to this work.

* Corresponding author:

Huafu Chen, The Clinical Hospital of Chengdu Brain Science Institute, MOE Key Laboratory for Neuroinformation, High-Field Magnetic Resonance Brain Imaging Key Laboratory of Sichuan Province, School of Life Science and Technology, University of Electronic Science and Technology of China, Chengdu, 610054, P.R. China. Email: [chenhf@uestc.edu.cn](mailto:chenhf@uestc.edu.cn).

Li Meng, Department of Radiology, Xiangya Hospital, Central South University, Changsha, 410008, P.R. China. Email: [mengli96130@csu.edu.cn](mailto:mengli96130@csu.edu.cn).

**Supplementary Tables**

**Supplementary Table 1 Clinical and demographic characteristics of HY score-specific subgroup comparisons.**

| Demographics | HC  (n = 70) | Stage Ⅰ  (n = 32) | Stage Ⅱ  (n = 41) | Stage Ⅲ  (n = 11) | Comparisons (*P*-value) | | |
| --- | --- | --- | --- | --- | --- | --- | --- |
|  | Mean ± SD | | | | Stage Ⅰ  *vs* HC | Stage Ⅱ  *vs* HC | Stage Ⅲ  *vs* HC |
| Gender  (male/female) | 38/32 | 19/13 | 22/19 | 6/5 | 0.631^a^ | 0.949^a^ | 0.987^a^ |
| Age (years) | 52.56 ± 10.92 | 56.06 ± 8.21 | 56.06 ± 8.21 | 51.18 ±12.50 | 0.177^b^ | 0.682^b^ | 0.681^b^ |
| Duration (years) | / | 6.91 ± 4.84 | 6.63 ± 4.86 | 12.73 ± 5.44 | / | / | / |
| Onset age (years) | / | 50.19 ±10.39 | 47.15 ± 12.68 | 40.64 ± 12.41 | / | / | / |
| Hoehn-Yahr | / | 1.67 ± 0.35 | 2.70 ± 0.25 | 4.46 ± 0.52 | / | / | / |
| UPDRS Ⅲ | / | 21.00 ± 9.64 | 36.87±14.40 | 56.27±13.26 | / | / | / |
| MMSE ^#^ | 27.12 ± 4.75 | 26.83 ± 2.94 | 26.94 ± 4.09 | 24.57 ± 2.64 | 0.233^b^ | 0.985 ^b^ | 0.002 ^b^ |
| MOCA ^#^ | 24.14 ± 5.88 | 23.67 ± 3.90 | 23.21 ± 4.41 | 19.2 ± 5.22 | 0.206 ^b^ | 0.119 ^b^ | 0.018 ^b^ |

Abbreviations: Values are presented as mean ± standard deviation. PD, Parkinson’s disease; HC, healthy controls; UPDRS, United Parkinson’s Disease Rate Scale.

^a^ χ^2^ test.

^b^ Nonparametric Mann-Whitney tests.

^#^ Partial score missed (MMSE: PD stage I, n= 29; stage II, n= 36, stage III, n= 7; HC, n= 52. MOCA: PD stage I, n= 18; stage II, n= 28, stage III, n= 5; HC, n= 49).

**Supplementary Table 2 Clinical and demographic characteristics of UPDRS Ⅲ score-specific subgroup comparisons.**

| Demographics | HC  (n = 70) | Stage Ⅰ  (n = 26) | Stage Ⅱ  (n = 32) | Stage Ⅲ  (n = 26) | Comparisons (*P*-value) | | |
| --- | --- | --- | --- | --- | --- | --- | --- |
|  | Mean ± SD | | | | Stage Ⅰ  *vs* HC | Stage Ⅱ  *vs* HC | Stage Ⅲ  *vs* HC |
| Gender  (male/female) | 38/32 | 12/14 | 22/10 | 14/12 | 0.479^a^ | 0.502^a^ | 0.969^a^ |
| Age (years) | 52.56 ± 10.92 | 55.46 ± 7.55 | 54.66 ± 11.44 | 52.31± 12.01 | 0.340 ^b^ | 0.410^b^ | 0.962^b^ |
| Duration (years) | / | 7.81 ± 5.85 | 6.28 ± 4.36 | 8.81 ± 5.56 | / | / | / |
| Onset age (years) | / | 48.00 ± 11.07 | 49.88 ± 11.80 | 43.92 ± 12.92 |  |  |  |
| Hoehn-Yahr | / | 1.81 ± 0.51 | 2.39 ± 0.56 | 3.44± 0.94 | / | / | / |
| UPDRS Ⅲ | / | 15.15 ± 5.25 | 31.31 ± 4.92 | 54.23 ± 9.62 | / | / | / |
| MMSE ^#^ | 27.12 ± 4.75 | 27.35 ± 3.16 | 27.11 ± 3.10 | 25.33 ± 4.32 | 0.841^b^ | 0.441^b^ | 0.018^b^ |
| MOCA ^#^ | 24.14 ± 5.88 | 23.14 ± 4.24 | 24.75 ± 3.08 | 20.76 ± 5.08 | 0.156^b^ | 0.652^b^ | 0.004^b^ |

Abbreviations: Values are presented as mean ± standard deviation. PD, Parkinson’s disease; HC, healthy controls; UPDRS, United Parkinson’s Disease Rate Scale.

^a^ χ^2^ test.

^b^ Nonparametric Mann-Whitney tests.

^#^ Partial score missed (MMSE: PD stage I, n= 23; stage II, n= 28, stage III, n= 21; HC, n= 52. MOCA: PD stage I, n= 14; stage II, n= 20, stage III, n= 17; HC, n= 49).

**Supplementary Table 3 Regions with reduced gray matter volume of overall group comparison in patients with PD.**

| Brain regions | MNI coordinates  (x,y,z) | Cluster size | *T*-value |
| --- | --- | --- | --- |
| L Caudate | -17 -3 20 | 2238 | -5.76 |
| L Hippocampus | -24 -9 -12 |  | -4.35 |
| L Putamen | -21 0 12 |  | -3.89 |
| R Caudate | 14 -2 23 | 724 | -5.47 |
| L Medial superior  frontal gyrus | 0 44 35 | 798 | -5.84 |
| L Superior frontal gyrus | -18 42 41 |  | -3.90 |
| L Superior temporal pole | -50 9 -6 | 389 | -4.57 |
| R Superior temporal gyrus | 53 -11 -12 |  | -4.20 |
| L Cerebellum | -8 -77 -29 | 676 | -4.64 |

Abbreviations: All clusters were one-tailed Gaussian random field theory (GRF) corrected for whole-brain cluster-level multiple comparisons (minimum z > 3.09, cluster significance p < 0.05 and voxel significance p < 0.001). Cluster size is reported in number of voxels and stereotaxic coordinates are reported in the Montreal Neurological Institute (MNI) standard space. L, left; R, right.

**Supplementary Table 4 Regions with reduced gray matter volume of the HY score-specific subgroup comparisons in patients with PD.**

| Stages | Brain regions | MNI coordinates  (x, y, z) | Cluster size | *T*-value |
| --- | --- | --- | --- | --- |
| Stage Ⅰ | L Caudate | -17 3 14 | 258 | -4.18 |
|  | L Cerebullum | -9 -78 -29 | 225 | -4.56 |
| Stage Ⅱ | L Caudate | -8 20 2 | 1313 | -5.56 |
|  | L Putamen | -18 18 0 |  | -4.38 |
|  | R Caudate | 14 -2 23 | 269 | -4.82 |
|  | L Angular gyrus | -48 -69 36 | 345 | -5.25 |
| Stage Ⅲ | L Hippocampus | -15 -8 -14 | 2053 | -5.31 |
|  | L Caudate | -6 11 -8 |  | -4.91 |
|  | L Parahippocampal gyrus | -29 -30 -12 |  | -3.97 |
|  | L Thalamus | -6 -21 17 | 588 | -4.65 |
|  | R Thalamus | 2 -14 8 |  | -4.6 |
|  | L Superior temporal pole | -50 15 -11 | 528 | -5.27 |
|  | L Middle temporal gyrus | -62 -41 2 | 615 | -4.98 |
|  | R Caudate | 11 15 -3 | 1822 | -4.57 |
|  | R Parahippocampal gyrus | 32 -32 -12 |  | -4.40 |
|  | R Hippocampus | 30 -33 -11 |  | -4.39 |
|  | L Fusiform | -26 -36 -15 | 206 | -4.11 |
|  | R Medial orbitofrontal cortex | 2 33 -12 | 481 | -5.13 |
|  | L Rectus | -9 27 -23 |  | -3.79 |

Abbreviations: All clusters were one-tailed GRF corrected for cluster-level multiple comparisons with cluster significance p < 0.05 and voxel significance p < 0.001 (minimum z > 3.09). Cluster size is reported in number of voxels and stereotaxic coordinates are reported in the Montreal Neurological Institute (MNI) standard space. L, left; R, right.

**Supplementary Table 5 Regions with reduced gray matter volume of the UPDRS Ⅲ score-specific subgroup comparisons in patients with PD.**

| Stages | Brain regions | MNI coordinates  (x,y,z) | Cluster size | *T*-values |
| --- | --- | --- | --- | --- |
| Stage Ⅰ | L Caudate | -14 2 23 | 293 | -4.30 |
|  | R Superior temporal gyrus | 50 -21 -5 | 287 | -4.31 |
| Stage Ⅱ | L Caudate | -17 3 14 | 2047 | -5.43 |
|  | L Putamen | -20 17 3 |  | -5.11 |
|  | R Caudate | 14 -2 23 | 565 | -4.92 |
|  | R Putamen | 24 8 11 |  | -4.03 |
| Stage Ⅲ | R Medial orbitalfrontal cortex | 0 33 -12 | 1176 | -5.68 |
|  | L Caudate | -8 20 -6 |  | -5.38 |
|  | L Medial orbitalfrontal cortex | -20 30 -18 |  | -4.16 |
|  | R Parahippocampal | 26 -36 -8 | 666 | -4.463 |
|  | R Fusiform | 26 -42 -11 |  | -4.43 |
|  | L Parahippocampal | 35 -48 -32 | 559 | -4.84 |
|  | L Fusiform gyrus | -29 -35 -20 |  | -4.28 |
|  | L Hippocampal | -27 -11 -15 | 389 | -4.66 |
|  | L Superior temporal pole | -50 15 -11 | 648 | -5.17 |
|  | L Superior temporal gyrus | -53 3 -14 |  | -4.22 |
|  | L Middle temporal gyrus | -62 -29 -3 | 563 | -4.97 |
|  | L Cerebellum | -32 -63 -54 | 471 | -4.32 |
|  | R Vermis | 6 -71 -44 | 599 | -4.04 |
|  | R Cerebellum | 33 -50 -38 | 405 | -4.39 |

Abbreviations: All clusters were one-tailed GRF corrected for cluster-level multiple comparisons (minimum *z* > 3.09, cluster significance was set to p < 0.05 and voxel p < 0.001). Cluster size is reported in number of voxels and stereotaxic coordinates are reported in the Montreal Neurological Institute (MNI) standard space. L, left; R, right.

**Supplementary Table 6 Caudate-associated CaSCNs in PD sequenced by HY scores.**

| Brain regions | MNI coordinates  (x, y, z) | Cluster size | *GC*  (*z* values) |
| --- | --- | --- | --- |
| L Caudate | -14 0 20 | 2708 | 12.91 |
| L Putamen | -23 6 2 |  | 12.45 |
| L Pallidum | -17 2 -2 |  | 14.28 |
| R Pallidum | 18 3 -2 | 2223 | 14.49 |
| R Caudate | 17 0 21 |  | 12.31 |
| R Putamen | 24 11 2 |  | 7.60 |
| L Thalamus | -11 -14 11 | 507 | 7.10 |
| R Anterior cingulate cortex | 9 44 5 | 918 | 6.17 |
| L Inferior temporal gyrus | -56 -24 -29 | 4401 | 12.82 |
| L Middle temporal gyrus | -50 5 -26 |  | 9.84 |
| L Middle temporal pole | -44 9 -33 |  | 4.72 |
| R Inferior temporal gyrus | 54 -21 -30 | 1433 | 9.68 |
| R Middle temporal gyrus | 62 -9 -24 | 1109 | 8.98 |
| L Lingual gyrus | -14 -77 -5 | 1180 | 7.16 |
| R Lingual gyrus | 17 -71 -3 | 1872 | 8.62 |
| L Precuneus | -12 -60 54 | 401 | 10.05 |
| R Postcentral gyrus | 35 -35 41 | 2393 | 11.53 |
| R Inferior parietal loulbe | 48 -50 47 |  | 7.39 |
| L Precentral gyrus | -20 -9 60 | 782 | 9.41 |
| L Superior frontal gyrus | -14 26 56 | 507 | 6.85 |
| R Superior frontal gyrus | 27 2 54 | 291 | 7.23 |
| R Triangular inferior frontal gyrus | 45 38 6 | 847 | 8.17 |
| R Middle frontal gyrus | 24 29 38 | 712 | 8.70 |
| L Middle frontal gyrus | -32 57 12 | 929 | 9.79 |
| R Supplementary motor area | 11 -5 65 | 320 | 9.48 |
| L Inferior frontal operculum | -41 8 26 | 315 | 7.09 |
| L Supramarginal gyrus | -57 -24 39 | 1957 | 9.63 |
| L Inferior parietal lobule | -51 -41 54 |  | 8.05 |
| L Middle occipital gyrus | -24 -65 35 | 510 | 7.43 |
| R Middle occipital gyrus | 30 -63 33 | 635 | 9.25 |
| R Superior occipital gyrus | 18 -90 18 | 207 | 8.17 |
| L Fusiform gyrus | -23 0 -44 |  | 5.60 |
| R Fusiform gyrus | 35 -8 -41 |  | 6.37 |
| L Cerebellum | -47 -50 -41 | 2814 | 8.93 |
| R Cerebellum | 9 -42 -21 | 223 | 6.09 |

Abbreviations: All clusters were one-tailed GRF corrected for cluster-level multiple comparisons (minimum *z* > 3.09, cluster significance was set to p < 0.05 and voxel p < 0.001). Cluster size is reported in number of voxels and stereotaxic coordinates are reported in the Montreal Neurological Institute (MNI) standard space. GC, Granger causality values; L, left; R, right.

**Supplementary Table 7 Caudate-associated CaSCNs in PD sequenced by UPDRS Ⅲ scores.**

| Brain regions | MNI coordinates  (x,y,z) | Cluster size | *GC*  (*z* values) |
| --- | --- | --- | --- |
| L Caudate | -17 -3 21 | 839 | 14.87 |
| L Putamen | -17 14 -6 | 315 | 5.19 |
| R Caudate | 12 11 -9 | 426 | 5.01 |
| R Putamen | 24 6 -11 |  | 4.64 |
| L Thalamus | -5 -14 8 | 955 | 8.61 |
| R Thalamus | 15 -26 8 | 244 | 6.42 |
| R Anterior cingulate cortex | 6 44 8 | 871 | 6.65 |
| L Inferior temporal gyrus | -53 -12 -29 | 2147 | 8.56 |
| L Superior temporal gyrus | -59 -12 0 | 1077 | 10.73 |
| L Middle temporal gyrus | -57 -11 -11 | 856 | 8.07 |
| R Middle temporal pole | 48 14 -35 | 672 | 8.24 |
| R Inferior temporal gyrus | 44 0 -39 | 1114 | 7.01 |
| R Middle temporal gyrus | 62 -36 -9 | 227 | 7.04 |
| R Lingual gyrus | 24 -87 -12 | 384 | 9.63 |
| R Angular gyrus | 42 -63 50 | 800 | 8.20 |
| L Superior frontal gyrus | -14 -11 77 | 782 | 9.06 |
| L Precentral gyrus | -23 -18 75 |  | 7.79 |
| R Supplementary motor area | 9 -6 63 | 210 | 7.43 |
| L Insula | -32 15 9 | 337 | 5.53 |
| L Postcentral gyrus | -50 -27 56 | 177 | 5.43 |
| R Postcentral gyrus | 42 -36 62 | 239 | 7.09 |
| R Inferior frontal orbital gyrus | 47 32 -5 | 314 | 9.12 |
| R Middle frontal gyrus | 32 36 29 | 634 | 10.84 |
| R Rolandic operculum | 45 -29 18 | 843 | 9.87 |
| R Precentral gyrus | 48 3 35 | 2020 | 9.08 |
| L Inferior parietal lobule | -32 -60 39 | 384 | 11.87 |
| R Inferior parietal lobule | 33 -50 42 | 244 | 6.76 |
| R Superior parietal lobule | 24 -51 71 | 271 | 6.09 |
| L Fusiform gyrus | -27 -44 -23 | 2138 | 11.69 |
| R Fusiform gyrus | 33 -68 -12 | 1629 | 10.79 |
| L Middle occipital gyrus | -38 -77 12 | 1190 | 13.32 |
| L Cerebellum | -47 -50 -41 | 1763 | 9.38 |
| R Cerebellum | 30 -47 -26 | 2082 | 7.94 |

Abbreviations: All clusters were one-tailed GRF corrected for cluster-level multiple comparisons (minimum *z* > 3.09, cluster significance was set to p < 0.05 and voxel p < 0.001). Cluster size is reported in number of voxels and stereotaxic coordinates are reported in the Montreal Neurological Institute (MNI) standard space. GC, Granger causality values; L, left; R, right.

**Supplementary Figures**

**
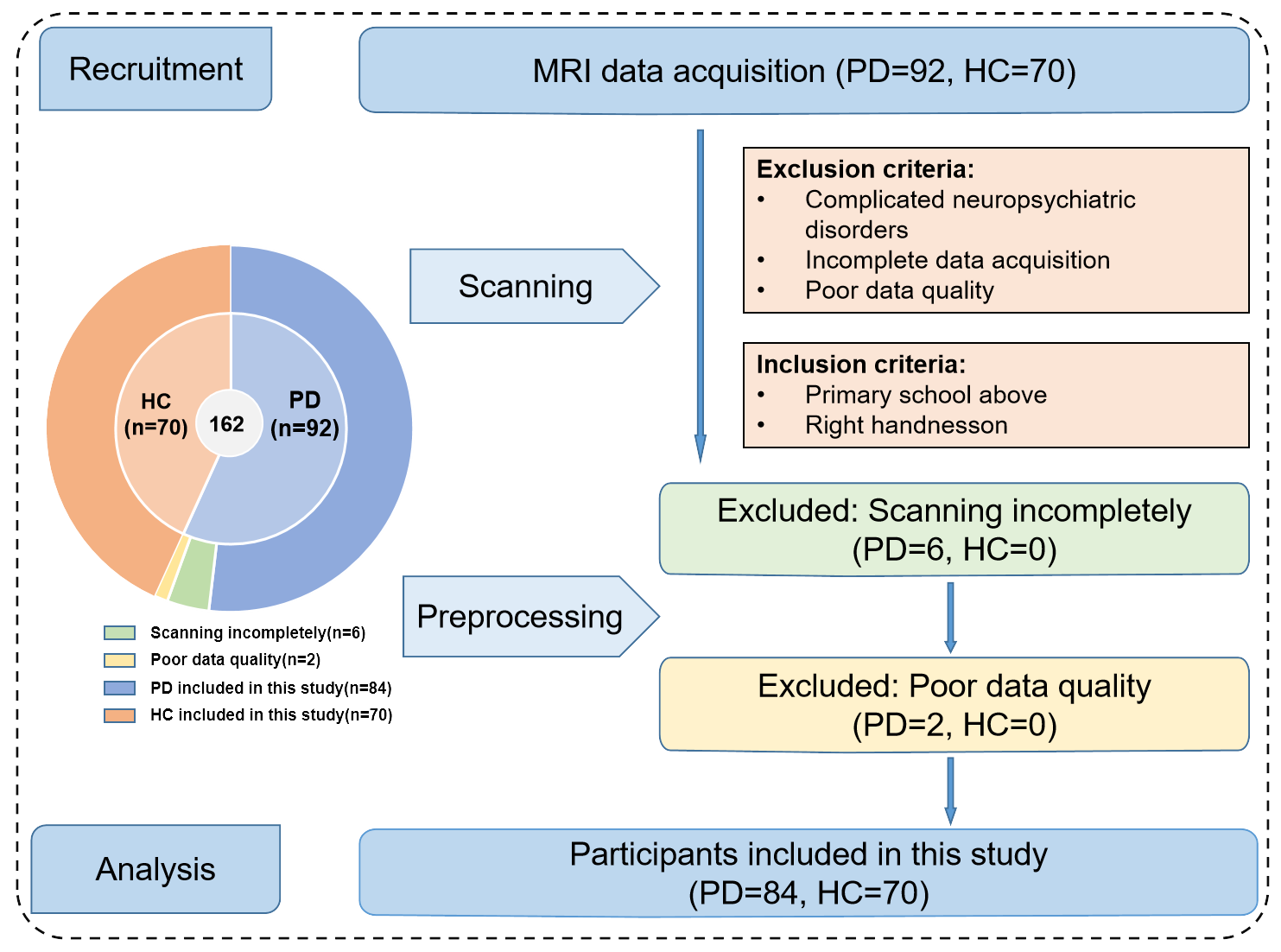
**

**Supplementary Figure 1. Procedure of inclusion and exclusion of the participants.** PD, Parkinson’s disease; HC, healthy controls.


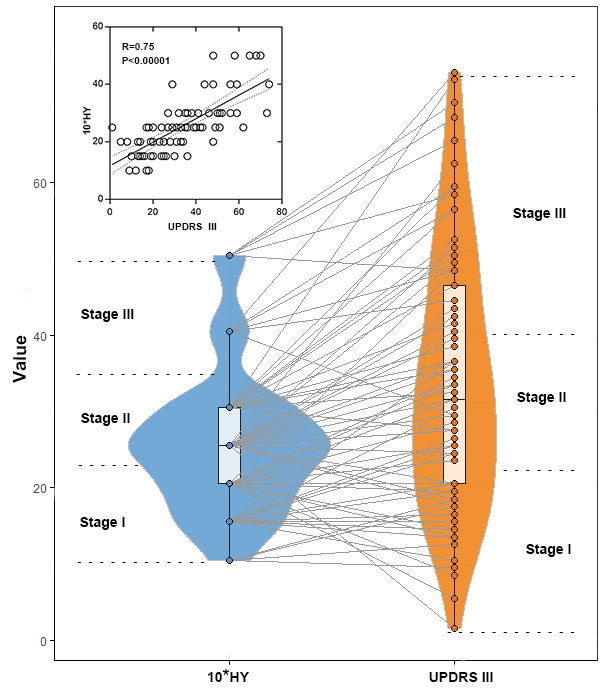


**Supplementary Figure 2.** A plot showing group membership across the three stages with paired points. The distribution of H&Y and UPDRS III scores was quite consistent at the stage level. Notably, significant relationship were found between H&Y and UPDRS III scores (r = 0.75, p < 0.00001).


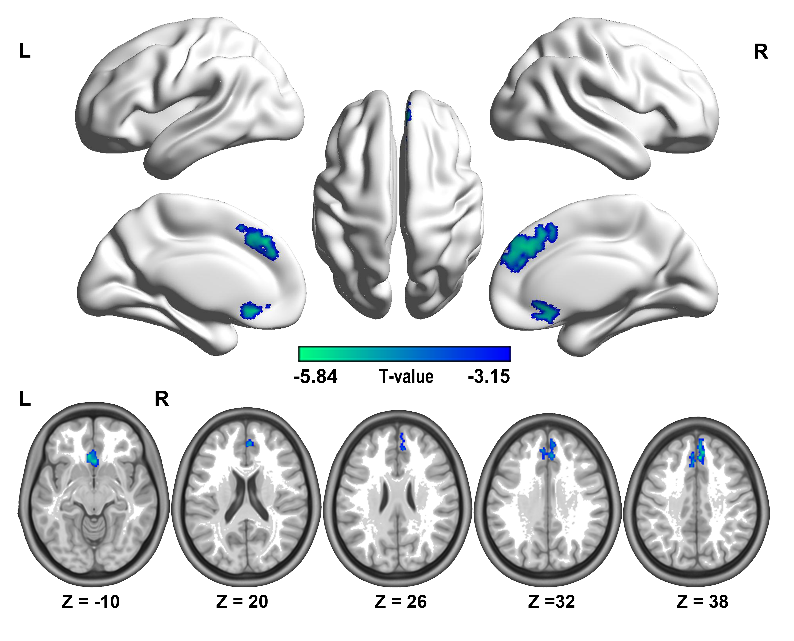


**Supplementary Figure 3.** Overall gray matter atrophy pattern between the PD patients with (n = 39) and without dementia (n = 12). Compared with the PD patients without dementia, patients with dementia displayed reduced gray matter volume in the left anterior cingulate cortex and right medial superior frontal gyrus. GRF was used for cluster-level correction for whole-brain multiple comparisons (minimum z > 3.09, cluster significance was set to p < 0.05 and voxel p < 0.001). L, left; R, right.
